# Hip fractures and area level socioeconomic conditions: a population-based study

**DOI:** 10.1186/1471-2458-9-114

**Published:** 2009-04-27

**Authors:** Andrea Icks, Burkhard Haastert, Manfred Wildner, Clemens Becker, Kilian Rapp, Nico Dragano, Gabriele Meyer, Joachim Rosenbauer

**Affiliations:** 1 Faculty of Public Health, Bielefeld University, Germany; 2 North-Rhine Westphalian Chamber of Physicians, Düsseldorf, Germany; 3 mediStatistica, Neuenrade, Germany; 4 Bavarian Health and Food Safety Authority, Oberschleissheim, Germany; 5 Department of Clinical Gerontology, Robert Bosch Hospital, Stuttgart, Germany; 6 Institute of Medical Sociology, Heinrich-Heine University of Düsseldorf, Düsseldorf, Germany; 7 Institute of Nursing Science, University Witten/Herdecke, Witten, Germany; 8 Institute of Biometrics and Epidemiology, German Diabetes Center, Düsseldorf, Germany

## Abstract

**Background:**

Only a limited number of studies have analyzed the association between hip fracture incidence and socioeconomic conditions. Most, but not all found an association, and results are in part conflicting. The aim of our study was to evaluate the association between hip fractures and socioeconomic conditions in Germany, from 1995 to 2004, on a census tract area level.

**Methods:**

We used data from the national hospital discharge diagnosis register and data on socioeconomic and demographic characteristics of 131 census tracts from official statistics. Associations between the hip fracture incidence and socioeconomic conditions were analyzed by multiple Poisson regression models, taking overdispersion into account.

**Results:**

The risk of hip fracture decreased by 4% with a 7% increase (about one interquartile range) of non-German nationals. It decreased by 10% with a 6% increased rate of unemployment, increased by 7% with a 2% increase of the proportion of welfare recipients, and also increased by 3% with an increase of the proportion of single parent families of 1.9%.

**Conclusion:**

Our results showed weak associations between indicators of socioeconomic conditions at area level and hip fracture risk; the varied by type of indicator. We conclude that hip fracture incidence might be influenced by the socioeconomic context of a region, but further analysis using more specific markers for deprivation on a smaller scale and individual-level data are needed.

## Background

Hip fractures cause substantial health deterioration and have a large economic effect due to the requirement of hospital and follow-up care [1,2]. The incidence of hip fractures increases with age, and the total number of fractures is expected to rise due to population aging.

All cause morbidity and mortality has been shown to be strongly associated with the individuals' socioeconomic position [3-6]. Besides individual-level analyses, a number of area-level analyses were performed, where the effect of a contextual deprivation, e.g. living in a low income neighborhood, were examined. Results show that apart from individual social characteristics, also characteristics of the social environment might impact health [7]. Possible pathways are the promotion of unhealthy lifestyles in deprived areas or an exposure to environmental health risks, such as an unhealthy build environment, air pollution, crime and others [8,9]. Thus, the evaluation of socioeconomic conditions in the individuals' environment is a complementary and more generalized concept of socioeconomic status. Both individual-level and regional-level socioeconomic indicators have been shown to be independently associated with the status of health [7,10].

Until now, only a few studies have researched hip fracture in relation to socioeconomic conditions at all, and there are also few studies at area level. Most of them found an increased hip fracture risk with social deprivation, whereas others did not [11-17]. Furthermore, one study found an increase for some indicators, and a decrease for other socioeconomic markers [18]. Thus, results are conflicting (table 1).

**Table 1:** Results from recent studies

**Study**	**Socioeconomic (SE) variable**	**Association between SE variables and hip fracture risk**
**Individual-level studies**		

Vestergard 2006, Denmark	- Social, co-morbidity variables;	Significant association
	- income	No association found
Peel 2007, Australia	- Psychosocial determinants of healthy ageing	Risk decrease

**Area-level studies**		

West 2004, UK	Townsend Score: social deprivation	No association found
Jones 2004, UK	Townsend Score: social deprivation	Risk increase
Kaastad 1998, Oslo (Norway)	- Urban vs. rural	Risk increase
	- poor socioeconomic conditions, high mortality	Risk increase
Sanders 2002, Australia	Urban vs. rural	Risk increase
Bacon 2000, USA	Lower income	Risk increase

**Individual- and area-level studies**		

Reimers 2007, Sweden	Individual-level:	
	-marital status: unmarried	Risk increase
	-county of birth: outside Sweden	Risk decrease
		
	Area-level:	
	- low economic status (high proportion of social welfare, unemployment, low-wage earners, single parent families)	Risk increase
	- low social status (high proportion of low educated subjects, high population density, low car ownership)	Risk decrease

Since the main concern of many research projects and preventive measures is the prevention of hip fractures under the elderly population [19-24], knowledge about socioeconomic conditions which might promote or prevent hip fracture may be helpful in tailoring more effective intervention programs [11].

The aim of our study was to estimate associations between hip fracture incidence and socioeconomic conditions in Germany (1995–2004) on a census tract area level, based on data of the hospital discharge register and population surveys.

## Methods

### Study design and population

This ecological study included data for the whole German population. It was based on the geographical level of 131 census tracts. Hip fracture incidences in the years from 1995 to 2004 were correlated with indicators of the socioeconomic conditions, measured between 1995 and 2001 at census tract levels.

### Data sources

To assess hip fractures, we used data from the national hospital discharge diagnosis register (Krankenhausdiagnosestatistik) from 1995 up to 2004. This register provides data about hospital discharges since 1993 and covers data from more than 99% of the hospitals in Germany. Each hospital discharge is registered with date, age and sex of the patient, patient's residence, and diagnosis. Diagnoses are coded using the International Classification of Diseases (ICD). Hip fractures were counted by ICD 9 diagnosis 820 (up to 1999) and thereafter by ICD 10 diagnosis S72.0, S72.1, and S72.2. A total of 3545 cases had to be excluded from the analyses because of missing values (0.29% of all 1214326 cases).

Regional socioeconomic and demographic characteristics were assessed at the level of 131 census tracts. Data on population, population density, percentage of welfare recipients, non-German nationals, unemployment rate, and total living space were taken from the annually updated official statistics covering the total population, provided by the Federal Statistical Office. Data on population income and proportion of single parent families were taken from census tract files. The German micro censorship is an annual survey of a representative 1% sample of all German households [25]. Definitions of regional level variables are presented in table 1.

### Statistical analyses

For all socioeconomic variables a weighted average was calculated from the calendar year-specific values available to give one respective figure for each of the 131 census tracts. From the national hospital discharge diagnosis register age/sex specific frequencies of hip fracture events were linked in each census tract to the socioeconomic variables. Age groups were 0–39, 40–59, 60–69, 70–79, 80+ years.

We estimated mean annual hip fracture incidences per 100,000 population, overall and stratified by age and sex in the census tracts, along with 95% confidence intervals, assuming Poisson distribution. To account for readmissions and double registrations, we used a correction factor of 0.89 which has been carefully evaluated and used in recent hip fracture incidence studies [26-29]. Incidences were age/sex standardized to the German population in 2000. The regional distributions of incidences were summarized by median, interquartile range (IQR), minimum and maximum. In a similar manner the socioeconomic variables were described. Additionally ratios of the interquartile range by the median were calculated (IQR ratio).

Spearman's rank-order correlation coefficients were used to investigate bivariate monotonic correlations between socioeconomic indicators. Associations between the hip fracture incidence and socioeconomic conditions were analyzed by "univariate age-sex adjusted" Poisson regression models, each including one socioeconomic indicator, sex, and age as independent variables. The estimated relative risks refer to changes of approximately the interquartile ranges (IQRs) of the social indicator. Furthermore, multiple Poisson regression models were fitted to investigate the simultaneous effect of the social factors, adjusted by sex and age (classified as above). Furthermore, to investigate age- or sex-specific associations, the analyses were performed stratified by sex or age classes. To account for overdispersion of incidence rates in Poisson regression, we performed all analyses using deviance adjusted variance estimates (SAS dscale-option) [30].

All analyses were performed using the Statistical Analysis Systems SAS (SAS for XP PRO, Release 9.1 TS1M3, SAS Institute Inc. Cary, NC, USA).

Since we used data from the official statistics, no permission of an ethical committee was needed.

## Results

### Population

The German population increased from 81.8 million in 1995 to 82.5 million in 2004. The proportion of younger inhabitants decreased (people aged 0–39 years: 52.3% in 1995, 46.8% in 2004), whereas the proportion of the higher age groups increased (proportion of people aged 70 or older: 10.6% in 1995, 12.3% in 2004).

### Incidences of hip fractures

The crude overall incidence of hip fractures in Germany in 1995–2004 was 131.0 (95% confidence interval 130.8–131.3) per 100,000 person years, and it was 130.0 (129.7–130.2) after standardization for the population in 2000. The median regional incidence (standardized to 2000) was 128.0, with an interquartile range of 12.7. There was a 1.6 fold difference in the hip fracture incidence between the area with the highest and that with the lowest incidence (162.2 versus 100.7 per 100,000 population).

### Socioeconomic indicators

The sociodemographic and socioeconomic variables showed marked differences between census tracts, as can be seen by the ratios of IQR and median (table 2). A pronounced difference was found for population density, and small variations for household income and living space. As expected, the various variables were highly correlated. A total of 17 correlations out of 21 were statistically significant (p < 0.05).

**Table 2:** Indicators of socioeconomic conditions based on official statistics, Germany

Socioeconomic indicator	Definition (unit of measure) ^a^	Median, interquartile range (IQR), Ratio of IQR and median, (minimum, maximum)
Total population ^b^ (1996–2000)	Total census tract population (N)	560802, 224900, 0.40 (124757, 3418720)
Census tract area ^b^	Census tract area (km^2^)	2531, 3337, 1.32 (78, 11542)
Population density ^b^ (1995–2000)	Number of people per km^2 ^ (persons per km^2^)	238, 643, 2.71 (57, 3917)
Non-German nationals ^b^ (1995–2000)	Non-German nationals in census tract population (%)	8, 7, 0.83 (1, 25)
Household income ^c^ (1991–2001)	Household income per no. of persons per household (Euro per person)	780, 102, 0.13 (609–1059)
Living space ^b^ (1996–2000)	Living space per no. of persons (m^2 ^per person)	38, 5, 0.13 (31–44)
Single-parent family ^c^ (1991–2001)	Single-parent families among all families (%)	8, 2, 0.23 (7–15)
Unemployment rate ^b^ (1996–2000)	Persons without a job among all persons capable of work (%)	10, 6, 0.64 (5–22)
Welfare recipients^b^ (1996, 1997, 2000)	Welfare recipients in census tract population (%)	3, 2, 0.69 (1–12)

^a ^For each indicator, a weighted average of calendar year-specific values was calculated at census tract level

^b ^Data from annually updated official statistics covering the total population ^c ^Data from census tract files (microcensus)

### Associations between hip fracture incidence and socioeconomic conditions

In univariate age/sex adjusted Poisson regression, all demographic and socioeconomic indicators except for the proportion of single parent families were significantly associated with hip fracture incidence, however, the risk ratios were close to 1 (table 3). In the multiple regression model, the direction of the association changed in part, and only the associations between hip fracture incidence and the proportion of non-German nationals, the unemployment rate and the proportion of welfare recipients remained significant. Additionally, the proportion of single parent families was significantly related to hip fractures in the multivariate model. The risk of hip fracture decreased by 4% with a 7% increase (about one interquartile range) of non-German nationals. It decreased by 10% with a 6% (about one interquartile range) increased rate of unemployment. It increased by 7% with a 2% increase of the proportion of welfare recipients (about one interquartile range), and also increased by 3% with an increase of the proportion of single parent families of 1.9% (about one interquartile range) (table 3).

**Table 3:** Relative risks of hip fracture according to categories of socioeconomic indicators, Germany, 1995–2004

Socioeconomic indicator	Relative Risk						
	Univariate Poisson regression ^a^	Multiple Poisson regression ^b^					

		Total	Sex-specific		Age-specific (years)		
	Total		male	female	<40	40–69	70+

Population density (per increase of 643 persons per km^2^)	1.03**	1.01	1.02**	1.01*	0.99	1.02*	1.01*
Living space per person (per decrease of 5 m^2 ^per person)	0.97**	1.00	0.99	1.01	0.97	0.96	1.01
Non-German nationals (per increase of 7%)	1.05**	0.96*	0.96*	0.96**	0.98	0.97	0.96**
Unemployment rate (per increase of 6%)	0.98**	0.91**	0.94**	0.90**	1.08	0.95	0.89**
Welfare recipients quota (per increase of 2%)	1.06**	1.07**	1.04**	1.08**	0.93**	1.04*	1.09**
Single parent families quota (per increase of 2%)	1.01	1.02**	1.03**	1.02**	1.02	1.02	1.03**
Household income per person (per decrease of 102 euro)	0.95**	1.00	1.00	0.99	1.03	1.01	0.99

^a ^Models including sex, age (0–39, 40–59, 60–69, 70–79, 80+ years), and each socioeconomic indicator separately

^b ^Model including sex and/or age, and all socioeconomic indicators * p < 0.05 ** p < 0.01

### Associations between hip fracture incidence and socioeconomic conditions by sex and age

There were no differences in the direction of the association between hip fracture risk and socioeconomic indicator between men and women. Sex-specific analyses revealed additionally a significant association between hip fracture risk and the population density, with an increased risk with a higher population density (table 3).

The associations between hip fracture incidences and sociodemographic and socioeconomic variables seem to differ between people aged younger than 40 years, and those aged 40 years and above. However, in the younger age group the associations were small and not statistically significant for most indicators. In the elderly, the associations did not differ substantially from the sex-specific results.

## Discussion

### Study findings

In our area-level analysis, we found only small differences in hip fracture incidence in relation to indicators of socioeconomic conditions. In multiple analyses, the hip fracture incidence was reduced with increased proportion of non-German nationals. It increased in regions with higher proportions of welfare recipients and single parent families, indicating a higher fracture risk in social deprived regions. However, a higher unemployment rate, an indicator for social deprivation, was associated with a lower hip fracture risk. In age- and sex-stratified analyses, in men and women as well as in the elderly, the hip fracture incidence was also higher with a higher population density. Overall, the risk ratios were close to 1, maybe in part due to large census tracts. Nevertheless, even when relative risks are small, attributable risks may be substantial due to a high exposure prevalence. Thus, we have to consider a considerable number of cases, indicating substantial relevance for community health.

### Comparison to other studies

Only few studies have evaluated the association between hip fracture incidence and socioeconomic position, and results were conflicting (table 1). Two studies evaluated this association on an individual level: In a Danish study, Vestergaard et al found associations between the hip fracture risk and several social and co-morbidity variables. However, they did not find an association to income [16]. In a study from Australia, psychosocial determinants of healthy ageing were found to be associated with a reduced risk of hip fractures [17]. Only one study investigated individual as well as environmental factors [18]. In this recent study in Sweden, the hip fracture risk was higher by unmarried individuals, and lower for those who were born outside of Sweden. Regarding environmental factors, the hip fracture risk was higher in regions with low economic status, defined as those with a high proportion of social welfare, unemployment, low-wage earners, and single parent families; however, there was a lower hip fracture risk in regions with low social status, defined as high proportion of low educated subjects, high population density, low car ownership, and high proportion of rented accommodations. [18]. Further studies investigated the association between the risk of hip fracture and socioeconomic conditions on a regional level. In a U.K. study, using the deprivation Townsend Score, no significant association between hip fractures and socioeconomic position of the area of residence was observed. However, the lack of significance may be due to low statistical power, since there was a statistically significant association between the risk of fall-related hospital admissions and the Townsend Score with a higher risk in deprived regions [11]. In a second UK study, also using the Townsend Score, a higher hip fracture risk was observed in regions with a higher proportion of socially deprived subjects [13]. Kaastad et al [12] found a higher risk of hip fractures in Oslo (Norway) compared to rural county. In the city of Oslo, Norway, the hip fracture incidence was higher in city areas with poor socioeconomic conditions and higher mortality. Also in Australia, hip fracture incidences were higher in urban than in rural communities [14]. In the US, the association between hip fracture incidence and social conditions was evaluated in an ecological study based on the distribution of the household income. There was a linear decrease in hip fracture risk with an increase of income level [15].

All except the study of West presented sex-specific results. Patterns of the relationship between socioeconomic conditions and the hip fracture incidence were similar in men and women. Only one of the studies also evaluated the associations in younger age groups: Marked effects of socioeconomic conditions were seen, whereas the effect diminished with age and was no longer observed in older age groups [13].

### Discussion of our results

A higher hip fracture risk may be considered to be related to an adverse social environment. In regions with a high level of social deprivation, the proportion of people with an unhealthy lifestyle, such as smoking, alcohol consumption, bad nutrition, low activity level, and poor use of health care and preventive services might be high [9]. All these conditions are risk factors for hip fractures [12,15]. Furthermore, a high deprivation level may reflect a poor local availability of resources like health care or preventive services [12,15]. The observed higher risk in regions with a higher welfare rate in our study is in line with these theoretical and empirical presumptions, although the reversed effect in younger age groups is unexpected. The lower hip fracture incidence with a higher unemployment rate in older age was also unexpected. The higher incidence with higher unemployment in younger age groups may be questionable. Age-dependent causes of hip fracture events might be considered. Hip fracture risk in younger age is related to manual labour, work at home, athletic injuries, and traffic accidents. A high unemployment rate in a region might be a marker for a more industrial area and therefore a higher number of workers with higher hip fracture risks. It might also be related to a higher alcohol consumption and subsequent traffic accidents in a young age group.

The inverse relationship between unemployment and hip fractures in higher age groups is more difficult to explain. The association at age 40–69 may be spurious. It may also be related to a more sedentary life style, less sport, dependence on public transport and less self-owned property requiring maintenance. The lower apparent risk at age 70+ may be an effect of residual confounding of an open age group: life expectancy in socially deprived groups is much lower, while hip fracture risk increases exponentially with age. A further possible explanation may be that in the Eastern part of Germany, where unemployment is higher compared to Western parts of Germany, the hip fracture risk is in general lower than in the Western regions [28,29] and hence unemployment rate is a proxy for the former East-West division. It could be hypothesized that the welfare rate might be a better indicator for social deprivation in the elderly than unemployment.

Regarding other SES indicators, the association between a higher hip fracture incidence and a higher population density is in line with earlier studies. These studies found an increased risk of hip fractures in urban compared to rural regions. A higher physical activity level in rural regions was assumed as a possible reason [14]. Another explanation may be a migration bias, since frail people at higher risk for hip fracture may be more likely to choose living in a city for reasons of health care and infrastructure [14]. A lower hip fracture risk in people of foreign nationalities has been observed in Scandinavian regions, too. Compared to middle European countries, lower incidences of hip fractures has been found in South Europe and in Asian and African populations, whereas incidences have been high in Northern European countries [31]. It remains unknown whether constitutional or lifestyle factors in subjects from other ethnic origin play a role [18]. Regarding the findings of our study it is noteworthy that there is a considerable difference in the age distribution of German and non-German inhabitants. The immigrant population is significantly younger due to work driven migration which could have influenced incidence rates in regions with a high proportion of non German nationalities. It would have been helpful to distinguish between different nationalities in our analysis, but such data was not available. However, the largest part of non-German inhabitants countrywide has Turkish origin (26%), followed by Italians and immigrants from Serbia/Montenegro (8 and 7%, respectively) [32]. Again, residual confounding due to the open age group 70+ is possible.

In general, differences between the age groups are plausible because the etiology of hip fractures varies by age. In younger ages, hip fractures are predominantly induced by major traumatic causes like car and working accidents or sport injuries, whereas hip fractures in the elderly are predominately related to osteoporosis and falls in combination with relatively minor external trauma. The associations between fracture incidence and socioeconomic conditions in the younger age group in our analysis by and large failed significance, maybe due to low power associated with a low incidence of hip fractures in this age group.

### Limitations

Several limitations of our study have to be addressed. (1) Assessment of hip fracture incidences might be biased by coding errors in the hospital discharge register. Additionally, the classification system changed during the observation period (ICD 9 to ICD 10). However, hip fractures are clearly categorized in both versions of the ICD. The German hospital discharge data have not been validated with respect to hip fractures. A study in the UK found an excellent accuracy and reliability of hospital-coded records when compared to prospective hip fracture data collection [33]. We assume that also in Germany the hip fracture diagnosis is valid, and the hospital discharge register has been used several times for epidemiological studies regarding the incidence of hip fractures [27-29,34]. (2) We used an actual correction factor to account for recurrent admissions. Although the factor has been carefully evaluated in Germany and used in previous studies [26-29], we cannot exclude that the number of recurrent hospital admissions differs by region. (3) Socioeconomic variables did not cover the whole period. However, we decided to take variables which had been used previously to evaluate morbidity and socioeconomic conditions in Germany [35]. Regional socioeconomic conditions were assessed from a broad spectrum of socioeconomic indicators taken from annually updated official statistics for the whole population and from a mandatory standardized household survey which is conducted every year (the German Micro Censorship). Thus, in contrast to other ecological studies, our socioeconomic data covered a reasonable time span. (4) A source of bias is the variation of social conditions within the observed regions. As the units of analysis (census tracts) were large, the risk of misclassification is high as the variability of socioeconomic condition inside the regions should be considerably high. (5) Results have to be interpreted with caution to avoid an "ecological fallacy" It could not be ruled out, that the observed statistical relations are artificial as we did not measure intermediate paths which might link socioeconomic conditions on regional level and risk. Nevertheless it can be assumed, that there are plausible links between the two levels. For example, it might be that the quality of the infrastructure in streets and parks is lower in socially deprived regions. These effects are not covered by studies at the individual level ("individualistic fallacy") [36,37]. Multilevel analyses have shown independent effects of individual and environmental levels of the socioeconomic position on health [7,10]. To our knowledge only one study has evaluated the association between hip fractures at individual and environmental level at the same time so far but results were conflicting [18]. The hospital discharge register does not provide individual data of the socioeconomic position. However, it provides the advantage of a nationwide complete data base for hip fractures over a long period. Thus, the strength of our study is the excellent population coverage of the data, available over a long time span.

## Conclusion

In this first analysis of the association between hip fracture incidence and socioeconomic conditions on regional level in Germany, we found small differences in hip fracture incidence with varying area level socioeconomic conditions, in comparison to several studies from other countries. This may be in part due to large census tracts. Our results may indicate a higher fracture risk with a higher welfare rate, a higher population density, and a lower risk in regions with a high proportion of non-German nationals. No associations were found for average income and results for unemployment showed an inverse relationship. Thus, hip fracture incidence might be influenced by socioeconomic context of a region, but further analysis using more specific markers for deprivation on a smaller scale are needed.

As the incidence of hip fractures is assumed to increase in the future due to population ageing [27-29,38-43], preventive interventions are required. Further ecological studies with a smaller scale of aggregation and large cohort studies are warranted to gain a more detailed insight in the association between hip fracture risk and social position on an individual level as well as socioeconomic conditions in the individual's environment. This would help to target prevention programs to individuals at risk and to implement protective environmental conditions, e.g. health care and preventive services.

## Competing interests

The authors declare that they have no competing interests.

## Authors' contributions

AI made the concept of the analysis and wrote the paper. BH did the data management and the statistical analysis. All co-authors (BH, MW, CB, KR, ND, GM, JR) discussed the concept and the results including substantial contributions and revised the paper critically. ND additionally gave special support to the socioeconomic questions, and JR provided the socioeconomic data. All authors participated in the work sufficiently to take public responsibility for appropriate portions of the paper.

## Authors' information

AI is working in the field of epidemiology and health in the elderly at the Institute of Epidemiology and International Public Health, Bielefeld University. BH is an expert in statistics in medicine and public health. MW has been working in the field of fall and hip fracture prevention for several years. CB is geriatrician and chair man for Germany in the European project ProFane (falls prevention network). KR is member of CB's working group. ND is an expert in the field of social inequality. GM has longstanding experience in the field of falls prevention as well as evidence based medicine. JR is an expert in methods of epidemiology.

## Pre-publication history

The pre-publication history for this paper can be accessed here:


